# First evidence for the presence of amino acid sensing mechanisms in the fish gastrointestinal tract

**DOI:** 10.1038/s41598-021-84303-9

**Published:** 2021-03-02

**Authors:** Jessica Calo, Ayelén M. Blanco, Sara Comesaña, Marta Conde-Sieira, Sofia Morais, José L. Soengas

**Affiliations:** 1 Laboratorio de Fisioloxía Animal, Departamento de Bioloxía Funcional e Ciencias da Saúde, Facultade de Bioloxía and Centro de Investigación Mariña, Universidade de Vigo, 36310 Vigo, Pontevedra Spain; 2 Lucta S.A., Innovation Division, UAB Research Park, Bellaterra, Spain

**Keywords:** Molecular biology, Systems biology

## Abstract

This study aimed to characterize amino acid sensing systems in the gastrointestinal tract (GIT) of the carnivorous fish model species rainbow trout. We observed that the trout GIT expresses mRNAs encoding some amino acid receptors described in mammals [calcium-sensing receptor (CaSR), G protein-coupled receptor family C group 6 member A (GPRC6A), and taste receptors type 1 members 1 and 2 (T1r1, T1r2)], while others [taste receptor type 1 member 3 (T1r3) and metabotropic glutamate receptors 1 and 4 (mGlur1, mGlur4)] could not be found. Then, we characterized the response of such receptors, as well as that of intracellular signaling mechanisms, to the intragastric administration of l-leucine, l-valine, l-proline or l-glutamate. Results demonstrated that *casr, gprc6a*, *tas1r1* and *tas1r2* mRNAs are modulated by amino acids in the stomach and proximal intestine, with important differences with respect to mammals. Likewise, gut amino acid receptors triggered signaling pathways likely mediated, at least partly, by phospholipase C β3 and β4. Finally, the luminal presence of amino acids led to important changes in ghrelin, cholecystokinin, peptide YY and proglucagon mRNAs and/or protein levels. Present results offer the first set of evidence in favor of the existence of amino acid sensing mechanisms within the fish GIT.

## Introduction

The gastrointestinal tract (GIT) plays a key role in the control of food intake and energy balance in mammals^1^. Thus, it is well established that cells within the intestinal epithelium possess several types of transporters and receptors (chemo- and mechano-receptors) able to sense the presence of nutrients (carbohydrates, lipids/fatty acids and proteins/amino acids) in the lumen and respond to such information with the release of signaling molecules^2^. Although three intestinal cell types (brush cells, enterocytes, and enteroendocrine cells—EECs) have been proposed to participate in nutrient sensing, EECs are the primary chemosensory cells within the GIT^3^. EECs respond to the sensing of nutrients with the secretion of different hormones, mainly ghrelin (GHRL), cholecystokinin (CCK), peptide tyrosine-tyrosine (PYY) and glucagon-like peptide-1 (GLP-1). These hormones, apart from acting locally modulating digestion and gut motility, can enter into the *lamina propria* and bind to their respective receptors on the vagal afferent nerve, thus sending information on energy status to the central nervous system (CNS)^2,4^. Peripheral information received by the CNS is then integrated, especially in the hypothalamus and the hindbrain, resulting in the production of key factors that either stimulate or inhibit food intake^5,6^. This communication system establishes the basis for the gut-brain axis^7,8^.

The mechanisms by which the mammalian GIT senses and responds to nutrients have become an area of increasing scientific interest over the last decades and, to date, several sensing mechanisms have been detected and characterized^2,3^. However, research on gut nutrient sensing mechanisms in fish is very scarce. Fish are highly dependent on protein to meet their metabolic requirements, relying primarily on amino acids rather than on carbohydrates as substrates for energy metabolism, especially in carnivorous species. Indeed, optimal dietary protein levels of fish are 50–300% higher than in terrestrial farm animals^9^. Therefore, it is logical to assume that gut amino acid sensing mechanisms are essential, and even more so in predominantly carnivore fish species. In mammals, sensing of luminal amino acids is mainly driven by some G protein-coupled receptors (GPCRs), as well as specific amino acid transporters^10^. GPCRs involved in amino acid sensing include the calcium-sensing receptor (CaSR), the G protein-coupled receptor family C group 6 member A (GPRC6A), the taste receptor 1 family (T1Rs) and the metabotropic glutamate receptors (mGluR) 1 and 4^10^. CasR is the main responsible for the detection of aromatic amino acids, such as l-phenylalanine and l-tryptophan. GPRC6A responds to many types of amino acids, especially small neutral amino acids (such as l-alanine, l-glycine and l-serine) and basic amino acids (such as l-lysine, l-arginine and l-ornithine)^3,10^. The T1R family consists of three different subtypes (T1R1, T1R2 and T1R3), among which T1R1 and T1R3 heterodimerize to sense (in most mammals except humans) most of the L-type amino acids (except tryptophan)^3,10,11^. Finally, mGluR1 and mGluR4, both class C G protein-coupled receptors, have more recently emerged as glutamate-sensing taste receptors^3,12^. As for transporters involved in amino acid sensing in the GIT, widely expressed on the cell membranes, they are in charge of monitoring the amino acids concentration and mediating extra- and intra-cellular amino acids exchange. These transporters couple the movement of amino acids with that of ions, including Na^+^, H^+^, K^+^ and/or Cl^−^, or of other amino acids by antiport^10^.

In fish, studies available to date have demonstrated the presence of transcripts for *tas1r2* and *tas1r3* (genes encoding T1r2 and T1r3, respectively) in the midgut of rainbow trout (*Oncorhynchus mykiss*)^13^, as well as *gpcr6a* and *casr* mRNAs in the GIT of Atlantic salmon (*Salmo salar*)^14^. Additionally, *gnai1* (gene encoding guanine nucleotide-binding protein G(I) subunit alpha, possibly the fish equivalent to mammalian gustducin, considering that teleost fish apparently lack an ortholog of this protein^15^) mRNAs have been detected in the rainbow trout midgut^13^. In grass carp (*Ctenopharyngodon idella*), abundance of *tas1r1* and *tas1r3* has been measured in intestine, with significant changes occurring during the transition from their carnivore to herbivore feeding habits^16^. Furthermore, immunoreactive cells expressing taste-signalling G protein subunits, G_αtran_ and G_αgust_, have been detected in stomach and intestine of common sole (*Solea solea*)^17^ and seabass (*Dicentrarchus labrax*)^18^. These studies reported that the density of these molecules was modulated by diet composition in sole, and that G_αtran_-immunoreactive gastric cells in the sea bass co-expressed GHRL^18^, which is consistent with their likely participation in nutrient sensing mechanisms.

With this background, this study aimed to identify and characterize amino acid sensing mechanisms in the GIT of the carnivorous fish rainbow trout (*Oncorhynchus mykiss*). This research complemented previous studies in which we characterized nutrient sensing systems involved in food intake control in central and peripheral (liver) tissues^19^, and particularly amino acid sensing systems in the hypothalamus and hindbrain in the same model species^20,21^. With this objective in mind, we first studied the putative presence and expression pattern of the different known mammalian amino acid sensors, as well as of key gut hormones, along the trout GIT. Then, we characterized the response of those receptors, intracellular signaling mechanisms and gut hormones, to the intragastric administration of l-leucine, l-valine, l-proline or l-glutamate. The results obtained allowed us to characterize, for the first time in fish, the response of amino acid receptors and gastrointestinal hormones to the presence of different amino acids in the intestinal lumen.

## Results

### Amino acid receptors mRNAs are present in the rainbow trout gastrointestinal tract

**Figure 1:**
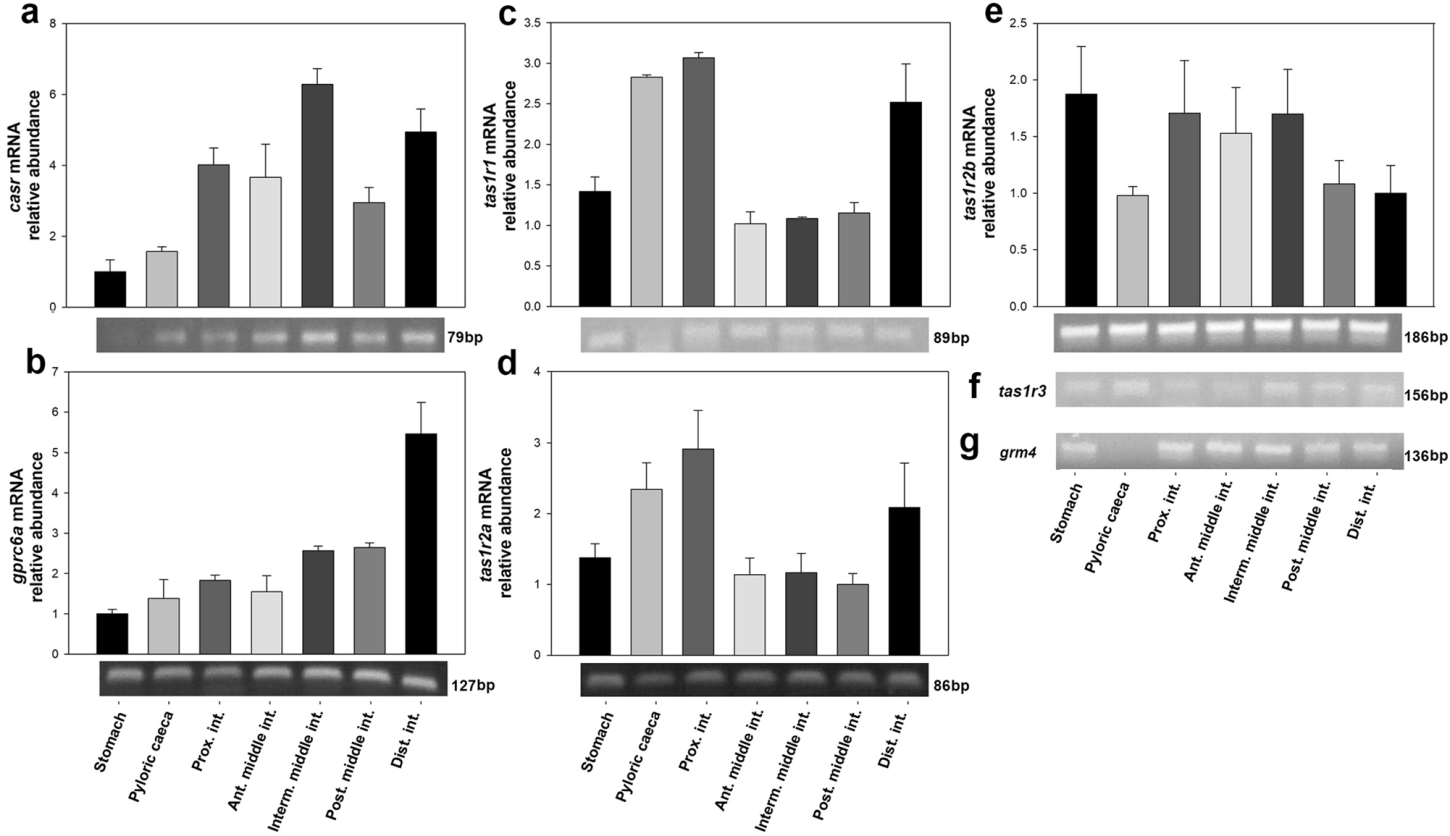
Distribution of amino acid receptors mRNAs along the rainbow trout GIT. For (**a**–**e**), quantitative analysis of mRNA abundance was performed by RT-qPCR considering *actb and ee1af1α* as reference genes. Data are expressed as mean + SEM (n = 6), relative to the tissue with the lowest mRNA abundance. For all genes, one sample from each tissue was run on a 1.5% agarose gel and representative bands are shown; please refer to Supplementary Fig. S1 for entire gels. *Ant. middle int.*, anterior middle intestine; *casr*, calcium-sensing receptor; *gprc6a*, *Dist. int.*, distal intestine; G protein-coupled receptor family C group 6 member A; *grm4*, metabotropic glutamate receptor 4; *Interm. middle int.*, intermediate middle intestine; *Post. middle int.*, posterior middle intestine; *Prox. int*., proximal intestine; *tas1r1*, *tas1r2a, tas1r2b*, *tas1r3*, taste receptor 1 family member 1, 2a, 2b, and 3.

As shown in Fig. 1 and Supplementary Figure S1, quantifiable levels of mRNAs encoding CasR, Gprc6a, T1r1 and two T1r2’s were detected, at different levels, in all of the studied regions of the rainbow trout GIT (stomach, pyloric caeca, proximal intestine, middle intestine and distal intestine). Specifically, *casr* was expressed predominantly in the intestine (in all areas but with higher levels in intermediate middle intestine and distal intestine), with very low levels of expression in pyloric caeca and almost undetectable levels in stomach (Fig. 1a). The expression of *gprc6a* tended to show an anterior–posterior gradient, with considerably higher levels in the distal intestine (Fig. 1b). Levels of *tas1r1* mRNAs were higher in pyloric caeca, proximal intestine and distal intestine, but it was present in all the GIT regions (Fig. 1c). Both *tas1r2a* and *tas1r2b* mRNAs were very abundant in the stomach, followed by the distal intestine and proximal intestine (*tas1r2a*) or proximal intestine and anterior plus intermediate middle intestine (*tas1r2b*) (Fig. 1d,e). Except for both *tas1r2* genes (expressed at relatively high levels), it is noteworthy that abundance of most receptors was low (yet quantifiable) throughout the GIT, as revealed by high Ct values (≈ 29–34) in the real-time PCR assays (data not shown). As for *tas1r3* and *grm4* (gene encoding mGlur4), corresponding transcripts were observed on agarose gels in most regions of the rainbow trout GIT, but their level of expression was minimal and therefore not possible to quantify accurately (Fig. 1f,g). Finally, neither conventional nor real-time PCR were able to amplify *grm1* in any of the regions of the GIT (data not shown).

### Luminal amino acids modulates amino acid receptors and intracellular signaling molecules mRNAs

**Figure 2:**
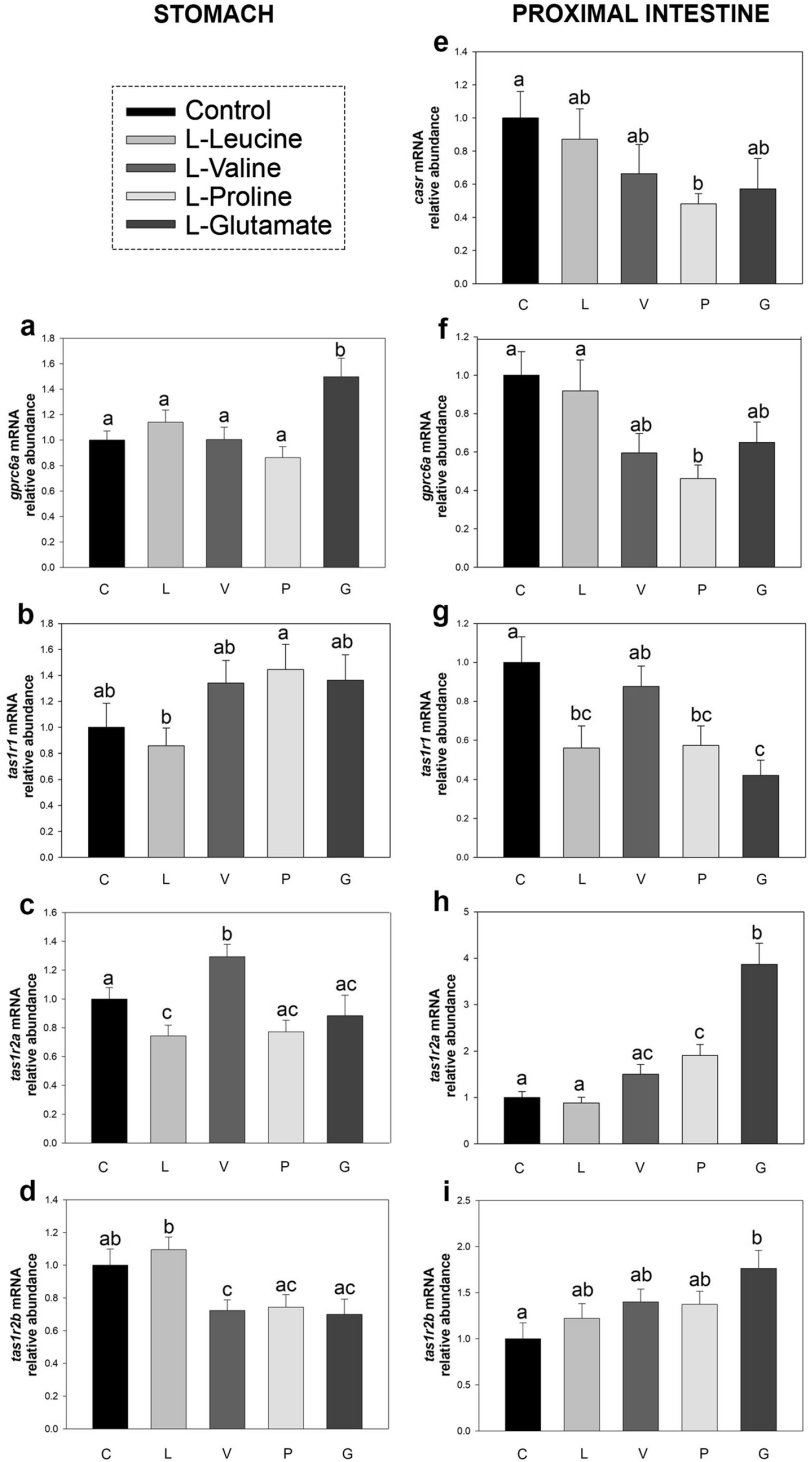
mRNA abundance of amino acid receptors in stomach (**a**–**d**) and proximal intestine (**e**–**i**) of rainbow trout 20 min after intragastric administration of 1 mL per 100 g^−1^ bw saline alone (control, C) or containing 40 μmol per mL^−1^ of l-leucine (L), l-valine (V), l-proline (P) or l-glutamate (G). Data obtained by RT-qPCR were normalized to the expression of *actb and ee1af1α* and are expressed as mean + SEM (n = 6), relative to the control group. Statistical differences among groups were assessed by one-way ANOVA and post-hoc Student–Newman–Keuls test using SigmaPlot software version 12.0. Significant differences are denoted by the use of letters: bars sharing the same letter are not statistically significant, while if two bars have different letters they are significantly different (p < 0.05). *casr*, calcium-sensing receptor; *gprc6a*, G protein-coupled receptor family C group 6 member A; *tas1r1*, *tas1r2a, tas1r2b*, taste receptor 1 family member 1, 2a, and 2b.

Figure 2 shows the effects of the intragastric administration of different amino acids on the mRNA abundance of amino acid receptors in the rainbow trout stomach and proximal intestine. Among the different amino acids tested, we observed that the luminal presence of l-leucine produced a significant decrease in the mRNA abundance of *tas1r2a* in the stomach (Fig. 2c) and of *tas1r1* in the proximal intestine (Fig. 2g). Administration of l-valine resulted in a significant increase in *tas1r2a* and significant decrease in *tas1r2b* transcripts in the stomach (Fig. 2c,d), with no effects in proximal intestine. l-proline affected the abundance of several receptors in the proximal intestine but not in stomach, downregulating *casr*, *gprc6a* and *tas1r1* (Fig. 2e,f,g) and upregulating *tas1r2a* (Fig. 2h). Finally, an increase in the abundance of *gprc6a* in stomach (Fig. 2a) and *tas1r2a* and *tas1r2b* in proximal intestine (Fig. 2h,i), as well as a decrease in *tas1r1* (Fig. 2g) in proximal intestine, were detected in response to the intragastric administration of l-glutamate.

**Figure 3:**
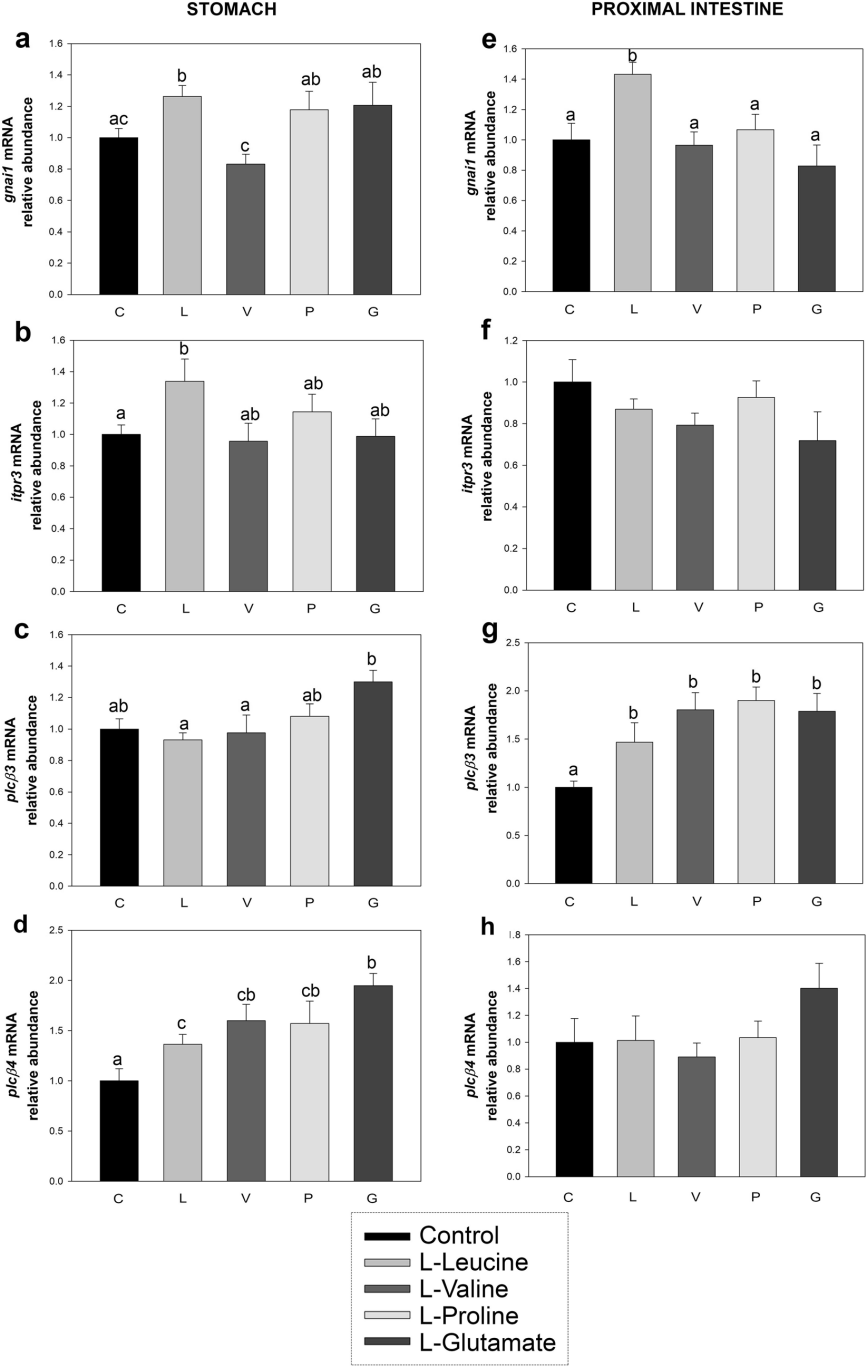
Effects of intragastric administration of 40 μmol per mL^−1^ of l-leucine (L), l-valine (V), l-proline (P) or l-glutamate (G) on the mRNA abundance of intracellular signaling elements in the rainbow trout stomach (**a**–**d**) and proximal intestine (**e**–**h**). Data obtained by RT-qPCR were normalized to the expression of *actb and ee1af1α* and are expressed as mean + SEM (n = 6), relative to the control group. Statistical differences among groups were assessed by one-way ANOVA and post-hoc Student–Newman–Keuls test using SigmaPlot software version 12.0. Significant differences are denoted by the use of letters: bars sharing the same letter are not statistically significant, while if two bars have different letters they are significantly different (p < 0.05). *gnai1*, guanine nucleotide-binding protein G subunit alpha 1; *itpr3*, inositol 1,4,5-trisphosphate receptor type 3; *plcb3, plcb4,* phospholipase C β3 and β4.

The luminal presence of amino acids also affected the mRNA abundance of intracellular signaling elements, as shown in Fig. 3. Thus, *gnai1* transcripts were found to increase at 20 min post-intragastric administration of l-leucine in the stomach (Fig. 3a) and proximal intestine (Fig. 3e). Abundance of *plcb3* mRNA was increased by l-glutamate in the stomach (Fig. 3b) and by all amino acids tested in the proximal intestine (Fig. 3f). On the other hand, all amino acids significantly upregulated *plcb4* mRNAs in the stomach (Fig. 3c), but no significant differences were observed in the proximal intestine (Fig. 3g). Finally, the luminal presence of l-leucine resulted in an increase in *itpr3* (gene encoding inositol 1,4,5-trisphosphate receptor type 3) transcripts in the stomach (Fig. 3d), while no changes were observed in proximal intestine after treatment with any of the tested amino acids (Fig. 3h). It is worth mentioning that we carried out several attempts to measure *plcb2* and *trpm5* mRNAs, but we were unable to amplify both transcripts in stomach or proximal intestine, as well as in any other region of the rainbow trout GIT, even if they can be detected in trout tongue (data not shown).

### Gut hormones are differentially expressed along the rainbow trout GIT

**Figure 4:**
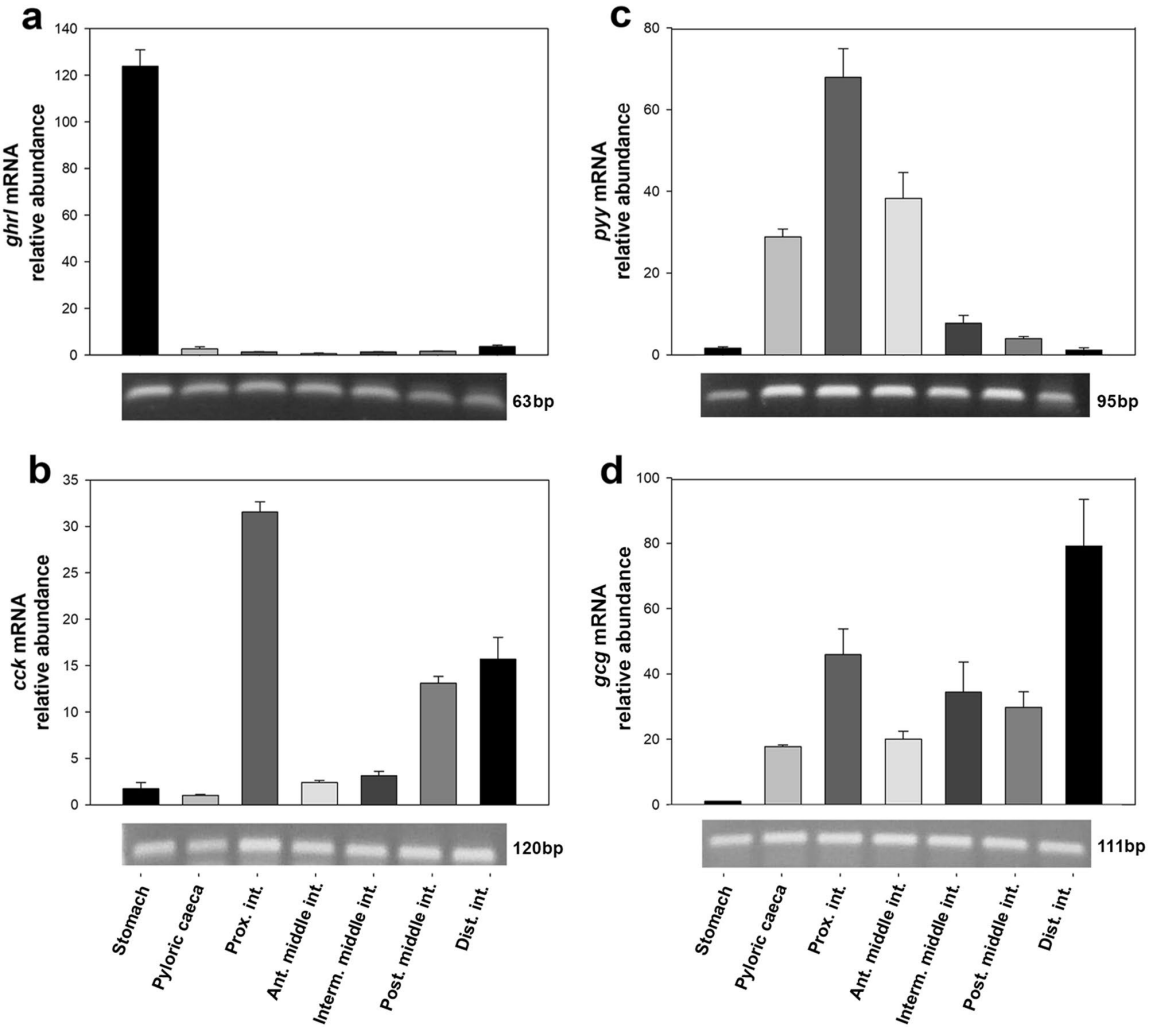
Distribution of gut hormones mRNAs along the rainbow trout GIT. Quantitative analysis of mRNA abundance was performed by RT-qPCR considering *actb* and *ee1af1* as reference genes. Data are expressed as mean + SEM (n = 6), relative to the tissue with the lowest mRNA expression. One sample from each tissue was run on a 1.5% agarose gel and representative bands are shown; please refer to Supplementary Fig. S1 for entire gels. *Ant. middle int.*, anterior middle intestine; *cck*, cholecystokinin; *Dist. int.*, distal intestine; *gcg,* proglucagon; *ghrl*, ghrelin; *Interm. middle int.*, intermediate middle intestine; *Post. middle int.*, posterior middle intestine; *Prox. int*., proximal intestine; *pyy*, peptide tyrosine-tyrosine.

Transcripts encoding the orexigenic peptide GHRL (*ghrl*) were found to be restrictively expressed in the rainbow trout stomach, with extremely low levels detected in the rest of the GIT regions (Fig. 4a and Suppl Fig. S1). On the contrary, the stomach showed very low abundance of mRNAs encoding the anorexigens CCK (*cck*), PYY (*pyy*) and GLP-1 (*proglucagon*, *gcg*), all of which were more abundant in regions of the intestine (Fig. 4b–d and Suppl Fig. S1). Specifically, *cck* mRNAs were predominantly observed in the proximal intestine, although considerable levels were also detected in the posterior middle intestine and distal intestine (Fig. 4b and Suppl Fig. S1). The proximal intestine, followed by the anterior middle intestine and pyloric caeca, also showed the highest abundance levels of *pyy* mRNAs. Levels of this transcript were very low in the intermediate and posterior middle intestine, and almost undetectable in the distal intestine (Fig. 4c and Suppl Fig. S1). Finally, *gcg* mRNAs were detected throughout the GIT except for the stomach, with the highest levels being measured in the distal intestine, followed by proximal intestine (Fig. 4d and Suppl Fig. S1).

### Gene and protein abundance of gut hormones respond to luminal amino acids

**Figure 5:**
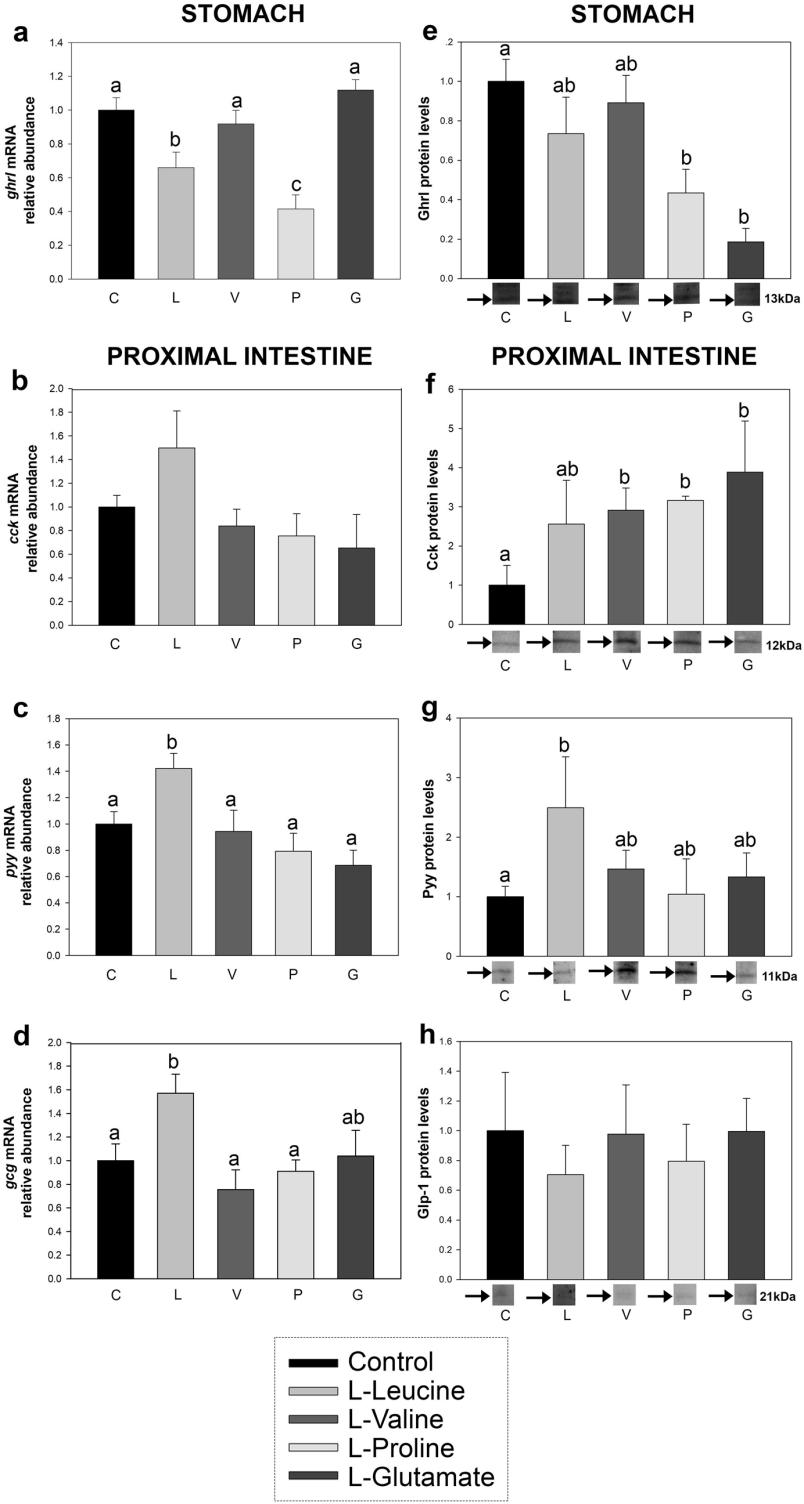
mRNA abundance of gut hormones (**a**–**d**) and proteins (**e**–**h**) in stomach (Grl) or proximal intestine (Cck, Pyy and Glp-1) of rainbow trout 20 min after intragastric administration of 1 mL per 100 g^−1^ bw saline alone (control, C) or containing 40 μmol per mL^−1^ of l-leucine (L), l-valine (V), l-proline (P) or l-glutamate (G). Data obtained by RT-qPCR (**a**–**d**) and Western blot (**e**–**h**) are expressed as mean + SEM (n = 6), relative to the control group. For (**a**–**h**), a representative band of each experimental group is shown for each protein; please refer to Supplementary Fig. S2 for entire blots. Protein bands were quantified by densitometry using Image Lab software, relative to the amount of total protein. Statistical differences among groups were assessed by one-way ANOVA and post-hoc Student–Newman–Keuls test using SigmaPlot software version 12.0. Significant differences are denoted by the use of letters: bars sharing the same letter are not statistically significant, while if two bars have different letters they are significantly different (p < 0.05).p Cck, cholecystokinin; *gcg*, proglucagon; Ghrl, ghrelin; Glp-1, glucagon-like peptide-1; Pyy, peptide tyrosine-tyrosine.

The intragastric administration of l-leucine and l-proline resulted in a significant downregulation of stomach *ghrl* mRNAs at 20 min post-administration (Fig. 5a). Likewise, both amino acids seemed to reduce Ghrl protein levels, but only significantly in the case of l-proline, while treatment with l-glutamate also significantly reduced protein levels (Fig. 5e and Supp Fig. S2). The mRNA abundance of *cck* in the proximal intestine was unaltered by the luminal presence of amino acids (Fig. 5b), but Cck protein levels tended to increase after treatment with all amino acids, although not significantly for l-leucine (Fig. 5f and Supp Fig. S2). Finally, the luminal presence of l-leucine caused a significant increase in both mRNA and protein abundance of Pyy, and in mRNA levels of *gcg* in the proximal intestine (Fig. 5c,d,g and Supp Fig. S2). Glp-1 protein levels in the proximal intestine were not affected by any of the amino acids tested (Fig. 5h and Supp Fig. S2).

## Discussion

The present study offered the first report on the putative functioning of amino acid sensing mechanisms in the GIT of an important aquaculture fish model, the rainbow trout. Firstly, we demonstrated that the rainbow trout GIT expresses mRNAs encoding key amino acid receptors described in mammals^10^, including CasR, Gprc6a, T1r1 and T1r2. Presence of *tas1r3* and *grm4* mRNAs was also detected but only at very low levels, insufficient to quantify accurately through real-time PCR. Except for CasR whose transcripts were measured at minimal levels in the stomach, all of the amino acid receptors quantified by real-time PCR were found in the rainbow trout stomach, proximal intestine, along the middle intestine and in distal intestine. The negligible expression of *casr* mRNAs in the stomach points to an important difference with respect to the mammalian model in which CasR, apart from being present in CCK-secreting I cells in duodenum and jejunum^22^, PYY and GLP-1-secreting L cells from duodenum up to colon^23^ and gastric inhibitory polypeptide (GIP)-secreting K cells in duodenum and jejunum^24^, is also importantly expressed in gastrin-secreting G cells of the stomach^25^. However, considering that here we are evaluating whole tissue, a lower amount of cells containing Casr in fish compared to mammals could explain such a difference. In addition, it is worth noting that the GIT differs among fish species and the proportion and function of its parts are very dependent of each species’ dietary habits. With regards to the other measured receptors, although this study does not enable a detailed assessment of position or colocalization of amino acid receptors with gut hormones, results are generally consistent with the distribution of mammalian counterparts along the mammalian GIT, where GPRC6A is preferentially expressed in intestinal and colonic L cells and at lower levels in gastric G cells^26^, and T1Rs receptor dimers involved in amino acid and carbohydrate sensing are mainly located in proximal intestinal CCK-secreting I cells and intestinal and colonic GLP-1 and PYY secreting L cells, respectively^27^. In these EECs, corresponding gut hormones are released in response to the sensing of amino acids. In mammals, these hormones send afferent signals to the CNS informing on energy status, and they also act locally modulating digestion and gut motility^2,4^. While knowledge on these pathways in fish is scarce, a role for Ghrl^28^ and Cck^29,30^ in the regulation of intestinal motility in zebrafish (*Danio rerio*), goldfish (*Carassius auratus*) and Atlantic cod (*Gadus morhua*) (respectively) has been described. In addition, Ghrl modulates the abundance of mRNAs encoding important digestive enzymes in the goldfish intestine^31^, and Cck stimulates the release of trypsin from the pyloric caeca of yellowtail (*Seriola quinqueradiata*)^32^, suggesting a role for these hormones on digestive processes in this species.

Concerning the distribution of transcripts for the different amino acid receptors along the trout GIT observed in this study, it is of special interest the relative high presence of *casr*, *gprc6a*, *tas1r1* and *tas1r2a* mRNAs in the distal intestine (compared to other GIT regions), region where we also detected higher levels of *cck* and *gcg* transcripts. A high abundance of *gcg* in the most posterior portion of the GIT is consistent with the mammalian model^33^, in which GLP-1 and PYY are predominantly expressed in the distal small and large intestine. Conversely, Pyy in fish seems to be expressed mostly in the anterior intestine, as suggested by this study and previous reports in Atlantic salmon^34^. As for CCK, it is released from I-cells (mainly in the upper small intestine in mammals^33^, thus partly differing from our observations showing a predominant abundance in proximal intestine but also an important presence in posterior middle intestine and distal intestine. The presence of Cck mRNA and protein has been previously reported in rainbow trout^35^, Atlantic salmon^34^ and white sea bream (*Diplodus sargus*)^36^ hindgut, where it was proposed to be involved in the feedback control of uncompleted digestive processes^36^. Considering our results, it might be plausible to hypothesize that conditions of uncompleted digestive processes would activate amino acid sensing mechanisms in the distal intestine, which would trigger the secretion of Cck to help optimize digestion and absorption. In favor of this hypothesis, Cck has been reported to increase contractions in the distal intestine of ballan wrasse (*Labrus bergylta*)^37^, facilitating digestive processes. Although some hypothesis could be drawn from the literature, the physiological importance of the existence of amino acid sensing mechanisms in the final region of the intestine is yet to be elucidated, and it is certainly an important subject for future research. Finally, results on the remaining assessed peptides in trout GIT is comparable to previous reports in fish^34^ and the widely accepted distribution of gut peptides in mammals^33^, with Ghrl being restricted to the stomach and GLP-1 predominantly expressed in the distal intestine.

Once the presence of amino acid receptors in the rainbow trout GIT was demonstrated, our next aim was to study aspects related to its functionality. For this, rainbow trout were intragastrically administered with l-leucine, l-valine, l-proline and l-glutamate to assess the transcriptional response of GIT amino acid receptors. It should be mentioned here that in this study we focused on the G protein-coupled receptors involved in gut amino acid sensing, and no amino acid transporter was measured. The levels of glucose and lactate in plasma displayed values comparable to those observed in unstressed individuals of the same species with no major differences observed among groups (Supplementary Fig. S3; https://osf.io/fxah8/), thus allowing us to suggest that no significant stress occurred in fish due to experimental conditions. However, since we did not evaluate plasma levels of cortisol and/or catecholamines we cannot exclude this possibility. Among the different amino acids tested, l-glutamate was found to exert the most significant changes in mRNA levels, modulating almost all receptors analysed. l-glutamate is the main amino acid responsible for the umami taste in mammals, being mainly sensed by T1R1-T1R3 and mGluRs 1 and 4^38^. Accordingly, mRNAs encoding all of these receptors have been reported to increase in the gastrointestinal tract of piglets after oral administration of monosodium glutamate^39^. As discussed above, neither T1r3 nor mGluRs were found expressed in the rainbow trout GIT, at least in sufficient amounts to be accurately quantified by the techniques employed here. However, l-glutamate-induced a significant upregulation of stomach *gprc6a* and proximal intestine *tas1r2a* and *tas1r2b*, and downregulation of proximal intestine *tas1r1* mRNAs, suggesting the involvement of Gprc6a, T1r1 and T1r2 in the detection of l-glutamate in the GIT of rainbow trout. In mammals, GPRC6A preferentially responds to basic and small neutral amino acids^3^, and therefore these results could indicate some differences in substrate specificity in trout. This is very likely the case of T1R2, which, heterodimerized with T1R3, is involved in the detection of carbohydrates in the mammalian gut^40^. However, in fish, T1r2′s-T1r3 heterodimers including multiple duplicated and functionally retained genes for *tas1r2*, have been demonstrated to respond mostly to amino acids, with an even broader activation profile than T1r1-T1r3^41^. This is probably related to the fact that energy metabolism in fish relies primarily on amino acids rather than on carbohydrates^42^, requiring a broad spectrum and enhanced sensitivity to amino acids. Present observation on l-glutamate significantly activating *tas1r2* mRNAs in the rainbow trout intestine is in line with the growing evidence supporting T1r2-T1r3 as an amino acid sensor in fish^41^. Similar to l-glutamate, the luminal presence of l-proline led to an increase in *tas1r2a* and a decrease in *tas1r1* mRNAs in the rainbow trout proximal intestine, suggesting a similar response of T1rs to amino acids with acidic side chains (l-glutamate) and cyclic amino acids (l-proline). This might not be too surprising, since results so far suggest that T1R receptors, in particular T1r2’s-T1r3 heterodimers, can be quite promiscuous in both zebrafish and medaka fish, thus responding to a wide range of amino acids^41^.

An interesting observation of our study is that several receptor genes responded in an unexpected way, through a reduction in their mRNA levels, in response to luminal levels of different amino acids. In addition to those already mentioned, l-proline induced a downregulation of mRNAs encoding Casr and Gprc6a in rainbow trout proximal intestine. In mammals it has been reported that both CasR (tuned by almost all l-amino acids) and GPRC6A are involved in the sensing of luminal l-proline^3,10,43^. However, while both receptors are activated in mammals^43^, l-proline seems to exert an inhibitory modulation in rainbow trout. In a similar fashion, we observed that the luminal presence of l-leucine decreased stomach *tas1r2a* and proximal intestine *tas1r1* mRNAs, and l-valine downregulated stomach *tas1r2b* transcripts. These results are certainly puzzling, and could indicate the existence of negative feedback loops involving different amino acids and receptors. However, research into this field in fish is still at too early stages to be certain of the physiological significance of these results or the mechanisms of action underlying such a response. Furthermore, we should keep in mind that amino acid sensing, through activation of receptors, occurs at the protein and not gene levels, and that studies on ligand specificity for most of these receptors, except for T1R’s in a reduced number of fish species, are still missing. Finally, it must be considered that only a short time post-administration of amino acids (20 min) was used to evaluate the response of nutrient sensors in this study; a longer time should be also tested to confirm the here observed responses and any putative additional response that may be triggered at a later time.

With the aim of delving into the intracellular signaling mechanisms triggered in response to the activation of amino acid receptors in the rainbow trout GIT, the mRNA abundance of key intracellular elements known to be involved in amino acid sensing in mammals was also quantified. While several signaling mechanisms have been reported in taste cells in mammals, the general model corresponds to the following: the binding of nutrients to GPCRs induces, via distinct G proteins (e.g., gustducin), the activation of PLC, resulting in the production of inositol triphosphate (IP_3_) and diacylglycerol (DAG). Then, IP_3_ binds to its receptor (ITPR3) in the endoplasmic reticulum (ER) membrane, releasing Ca^2+^ from the ER. The elevation of intracellular Ca^2+^ triggers the activation of TRPM5, which induces the depolarization of the cells, ultimately leading to the release of neurotransmitters or gut hormones in oral or GIT tissues, respectively^44,45^. Compared to this model, some important differences were noted in rainbow trout. First, it has been described that teleost fish do not have an ortholog of the mammalian gustducin gene (*gnat3*, guanine nucleotide-binding protein g(t) subunit alpha-3), but other G proteins, such as Gnai1, could be involved in gut sensing signaling instead^15,46^. The herein observed upregulation of *gnai1* abundance in both stomach and proximal intestine in response to the intragastric administration of l-leucine supports this hypothesis. On the other hand, while PLCβ2 appears to be the PLC subtype activated by nutrients in mammals^47^, none of the attempts made in the present study to identify *plcb2* transcripts in the rainbow trout GIT were successful, thus suggesting that this PLC subtype is not expressed in the GIT of this species. This is in contrast to its presence in oral tissues of the same^46^ or other fish species^48^, suggesting a tissues specificity in the expression of PLC subtypes. Instead, transcripts encoding Plcβ3 and Plcβ4 were found to be importantly expressed throughout the GIT of rainbow trout, and transcriptionally regulated by all tested amino acids, in either stomach or proximal intestine. In addition, we were also not able to detect the expression of *trpm5* in rainbow trout GIT despite several attempts, even though we have previously measured mRNA levels of this transcript in trout oral tissues^46^. In mammals, while the PLCβ2-mediated pathway is the most widely accepted signal transduction pathway involved in sweet, bitter, and umami taste detection in taste bud cells, it has been demonstrated that other alternative pathways independent of PLCβ2 and TRPM5 are also activated in response to these compounds. This evidence is based on genetic studies showing that the deletion of signaling proteins, including PLCβ2, ITPR3 and TRPM5, diminishes but does not completely abolish the detection of all bitter, sweet and umami stimuli^44,49–52^. Among the alternative signaling molecules described so far as being involved in taste transduction are PLCβ3^49,53^ and inositol 1,4,5-trisphosphate receptor type 1 (ITPR1)^53^. Present results showing the activation of *plcb3* and *plcb4* mRNAs in the rainbow trout GIT in response to the luminal presence of amino acids are in accordance with these observations in mammals, and point towards PLCβ3 and 4 being the main PLC subtypes involved in taste transduction pathways in the gut of this species. On the other hand, the few changes measured in the mRNA levels of *gnai1* and *itpr3* in stomach and proximal intestine, responding significantly only to l-leucine, could indicate the presence of alternative signaling pathways responding to amino acids in rainbow trout GIT.

As the final step of intracellular mechanisms triggered by amino acids in the intestinal lumen, the last aim of this research was to investigate the putative release of gastrointestinal hormones. Major results from this study demonstrated an amino acid-specific modulation of the abundance of Ghrl, Cck and Pyy mRNA and/or protein levels, and *gcg* mRNAs, in the rainbow trout GIT. Ghrl and Cck appeared to respond to all or almost all of the tested amino acids, with significant decreases in mRNA (after l-leucine and l-proline treatment) and protein (after administration of l-proline and l-glutamate, and only tendentially in the case of l-leucine) levels of Ghrl in stomach, and significant increases in Cck protein levels in proximal intestine after treatment with l-valine, l-proline and l-glutamate (non-significant increase in the case of l-leucine). In mammals, intraduodenal treatment with leucine and exposure of STC-1 enteroendocrine cells to individual l-amino acids (leucine, phenylalanine, tryptophan and glutamate) have been shown to increase plasma levels of CCK^54^ and stimulate CCK secretion^55^, respectively, while proline treatment did not affect plasma levels of CCK^54^. On the other hand, the activation profile of *pyy*/Pyy (mRNA and protein abundance) and *gcg* (mRNA) in rainbow trout proximal intestine was more restricted, responding significantly only to l-leucine. Interestingly, l-leucine was also the amino acid inducing highest changes in previous studies on rainbow trout focusing on hypothalamic amino acid sensing systems after ICV and IP treatment with amino acids^20,21^. Furthermore, both central and IP injections of l-leucine caused a clear anorexigenic effect in rainbow trout^20,21^, while it proved to be highly attractive to this species, significantly increasing the consumption of agar pellets containing this amino acid^21^. It can therefore be concluded that l-leucine has a key role in the control of food intake in trout, from the periphery to the brain.

In summary, the present study offers the first set of evidence suggesting that intestinal cells of rainbow trout respond to the luminal presence of amino acids with a modulation of gut hormones gene and protein abundance. However, it must be kept in mind that gene and protein levels do not always correlate with hormone release, and thus additional experiments (e.g., study of the gut hormone release in bloodstream following intragastric administration of amino acids) need to be done to verify release. The data presented suggests the existence of amino acid sensing mechanisms within the fish gut, with some similarities, but also differences, with respect to the widely accepted mammalian model. Important differences might relate to phylogenetical reasons (divergence between fish and mammals) and/or differences in dietary habits resulting from comparing a carnivorous fish model with omnivorous models (rodents, pig, human), but further studies, preferably on a variety of fishes (there are about 33,000 fish species, representing almost half of all vertebrate species), are required to elucidate the basis for these differences. Unraveling gut nutrient sensing mechanisms is essential for completely understanding the functioning of the gut-brain axis and the mechanisms governing the regulation of food intake and energy expenditure in fish.

## Material and methods

### Animals

Rainbow trout (*Oncorhynchus mykiss*; body weight (bw) = 100 ± 20 g), obtained from a local fish farm (A Estrada, Spain), were housed in five 100 L tanks (n = 40 fish/tank) containing aerated and dechlorinated tap water at 15 ± 1 ºC in an open circuit. Photoperiod was maintained at 12 h light:12 h darkness (12L:12D; lights on at 08:00 h). Fish were fed once daily at 11:00 h with commercial dry pellets (proximate analysis: 44% crude protein, 2.5% carbohydrates, 21% crude fat, and 17% ash; 20.2 MJ kg^−1^ of feed; Biomar, Dueñas, Spain) until visual apparent satiety. All studies adhered to the ARRIVE Guidelines, and were carried out in accordance with the guidelines of the European Union Council (2010/63/UE), and of the Spanish Government (RD 53/2013) for the use of animals in research, and were approved by the Ethics Committee of the Universidade de Vigo.

### Experimental designs

#### Study of amino acid receptors and gut hormones mRNA distribution along the GIT

Three fish were fasted for 48 h, anaesthetized in water containing 2-phenoxyethanol (0.02% v/v; Sigma-Aldrich, Madrid, Spain) and sacrificed by decapitation. The following regions of the GIT were dissected: stomach, pyloric caeca, proximal intestine, anterior middle intestine, intermediate middle intestine, posterior middle intestine and distal intestine (see Supplementary Fig. S4 for graphical details; https://osf.io/fxah8/). Samples were snap-frozen using dry ice and stored at − 80 ºC until quantification of the mRNA abundance of amino acid receptors and gut hormones along the trout GIT (see protocol below). This experiment was repeated twice.

#### Study of the response of amino acid receptors to the luminal presence of amino acids

This experiment was carried out during two days. Each day, thirty 48 h-fasted fish were captured from the acclimation tank in batches of six fish (accounting to n = 6 per condition each day), slightly anaesthetized with 2-phenoxyethanol (0.02% v/v), and individually weighed. Then, each of the five fish was intragastrically administered with 1 mL per 100 g^−1^ bw of one of the following treatments (randomly selected): saline solution (0.6% NaCl) alone (control) or containing 40 μmol per mL^−1^ of l-leucine, l-valine (both from Sigma-Aldrich), l-proline (Scharlau, Barcelona, Spain) or l-glutamate (Sigma-Aldrich). Experimental procedures started at 10:00 h each day and lasted for about 4 h. Administration was performed using a 13 cm-long cannula attached to a blunt-tip syringe. Putative regurgitation was monitored visually, and none was observed during administration of the different solutions. The dose of amino acids was calculated based on the amount of leucine (selected because it is the main amino acid acting on appetite regulatory mechanisms in the brain^56^) ingested per day by a trout fed a standard commercial diet^57^. An equimolar dose was used for the rest of amino acids. After administration, fish were allowed to recover in individual small tanks. Following 20 min, fish were again anaesthetized and samples of blood were collected by caudal venous puncture to obtain the plasma (by subsequent centrifugation for 10 min at 5000*g*). Then, fish were sacrificed by decapitation, and samples of stomach and proximal intestine were collected and immediately frozen using dry ice until further analysis of mRNA levels of amino acid receptors, intracellular signaling elements and gut hormones, and protein levels of gut hormones (see below). Plasma samples of all 12 fish per treatment were used for assessment of metabolite levels, while tissues from 6 fish per treatment were each used for RT-qPCR and Western blot analysis. Twenty min was selected as sampling time because preliminary experiments (not shown) demonstrated this time to be the necessary for a saline solution containing a dye to reach the intestine when administered intragastrically.

### Assessment of plasma metabolite levels

Levels of glucose and lactate in plasma (n = 12) were measured by enzymatic determination using commercial kits (Spinreact, Barcelona, Spain). Total α-amino acids were assessed using the colorimetric nynhydrin method^58^ with alanine as standard.

### Quantification of mRNA abundance by real-time PCR

Total RNA was isolated from tissues (n = 6 fish) using Trizol reagent (Life Technologies, Grand Island, NY, United States), and treated with RQ1-DNAse (Promega, Madison, WI, United States) following manufacturer’s instructions. RNA purity was tested by optical density (OD) absorption ratio (OD 260 nm/280 nm) using a NanoDrop 2000c (Thermo, Vantaa, Finland), and only samples with an OD 260 nm/280 nm ratio > 1.8 were used for analysis. Synthesis of cDNA was performed with 2 μg of total RNA using Superscript II reverse transcriptase (Promega) and random hexamers (Promega) in a final volumen reaction of 20 μL, according to the manufacturer’s instructions. Finally, gene abundance was quantified by real-time quantitative PCRs using MAXIMA SYBR Green qPCR Mastermix (Life Technologies). PCRs were carried out in 96-well plates loaded with 1 µg of cDNA and 750 nM of each forward and reverse primer in a final volume of 15 µL; duplicate wells were used for each sample. Sequences of primers used for target and reference genes (all ordered from IDT, Leuven, Belgium) are shown in Table 1. Primers used to study the presence of *grm1* (gene encoding mglur1), *plcb2* (gene encoding Plcβ2) and *trpm5* (gene encoding Trpm5, transient receptor potential cation channel subfamily M member 5) in the GIT are included in Supplementary Table S1 (https://osf.io/fxah8/). Such primers did not allow the amplification of neither *grm1*, *plcb2* and *trpm5* in the trout GIT, despite the fact that all of them amplify the target products in other tissues (rainbow trout tongue^46^), Negative controls were included in all reactions, consisting in wells containing RNA samples and water instead of cDNA. RT-qPCR cycling conditions consisted of an initial step of 95 ºC for 10 min, and 40 cycles of 95 ºC for 30 s and specific annealing and extension temperature (Table 1) for 30 s. Specificity of the amplification reaction was tested by a melting curve systematically monitored (temperature gradient at 0.5 ºC/5 s from 55 to 95 ºC) at the end of each run. Efficiency of all qPCR reactions was 95–100% and R^2^ was 0.97–1. All PCRs were run in an iCycler iQ (Bio-Rad, Hercules, CA, United States). PCR products were resolved on 1.5% agarose gels to confirm the amplification of a single product of the expected size. For the tissue distribution study, representative samples of each tissue were also run on 1.5% agarose gels. The 2−ΔΔCt method^59^ was used to calculate the relative abundance of target transcripts, using *actb* (gene encoding β-actin) and *ee1af1* (gene encoding elongation factor 1α), both stably expressed in this experiment, as reference genes.

**Table 1:** Primers used for determining gene expression in this study.

Gene	Data base	Accession number	Forward primer (5′ to 3′)	Reverse primer (5′ to 3′)	Annealing temperature (ºC)
*actb*	GenBank	NM 001124235.1	GATGGGCCAGAAAGACAGCTA	TCGTCCCAGTTGGTGACGAT	59
*casr*	GenBank	XM_021591413.1	TCGCCAGTGGGCCTGACATT	CTGGCCAACCAGATGCGGTC	60
*ccka*	GenBank	NM_001124345.1	GGGTCCCAGCCACAAGATAA	TGGATTTAGTGGTGGTGCGT	60
*eef1a1*	GenBank	AF498320	TCCTCTTGGTCGTTTCGCTG	ACCCGAGGGACATCCTGTG	59
*gcg*	GenBank	NM_001124698.1	AGGAGTGGTGCTCCATCCAAA	TCCTGATTTGAGCCAGGAAACA	59
*ghrl*	GenBank	AB096919.1	GGTCCCCTTCACCAGGAAGAC	GGTGATGCCCATCTCAAAAGG	60
*gnai1*	Sigenae	CU073912	GCAAGACGTGCTGAGGACCA	ATGGCGGTGACTCCCTCAAA	60
*gprc6a*	GenBank	XM_021574849.1	ATGGGGATCAGCAGAATTTGG	CCGGCACCTTGTTTCTCTTTG	60
*grm4*	GenBank	XM_021616324.1	CGCATCTTTGAGCAGGGGA	GAGACGGGTCAACACCAAACC	60
*itpr3*	GenBank	XM_021616029.1	GCAGGGGACCTGGACTATCCT	TCATGGGGCACACTTTGAAGA	59
*plcb3*	GenBank	XM_021577635.1	ATAGTGGACGGCATCGTAGC	TGTGTCAGCAGGAAGTCCAA	60
*plcb4*	GenBank	XM_021600840.1	ACCTCTCTGCCATGGTCAAC	CGACATGTTGTGGTGGATGT	60
*pyy*	GenBank	XM_021557532.1	GGCTCCCGAAGAGCTGGCCAAATA	CCTCCTGGGTGGACCTCTTTCCA	60
*tas1r1*	GenBank	XM_021614415.1	GTTGTGTTCTCCAGCAAAAGC	TCTGTCCCTATCCACACCTTG	60
*tas1r2a*	GenBank	MT240253	ATAGTTTTTGCCGGGCAGAGC	CCTGCAATCCACACTTTGCTG	59
*tas1r2b*	GenBank	XM_021625831.1	GATGAGTGGGCCAGGAATGG	CCTCCCACCGGCTGACTTTA	59
*tas1r3*	GenBank	XM_021569424.1	GCCCTGTGGAGCCCATCTTA	CCACACAGTAGGTCAGGGTGGA	60

*actb*, β-actin; *casR*, calcium-sensing receptor; *ccka*, cholecystokinin a; *eef1a1*, elongation factor 1α; gcg proglucagon; *ghrl*, ghrelin; *gnai1*, guanine nucleotide-binding protein G subunit alpha 1; *gprc6a,* G protein-coupled receptor family C group 6 member A; *grm4*, metabotropic glutamate receptor 4; *itpr3*, inositol 1,4,5-trisphosphate receptor type 3; *plcb*, phospholipase C-β; *pyy*, peptide tyrosine-tyrosine; *tas1r1/tas1r2a/tas1r2b/tas1r3*, gene encoding taste receptor 1 family members 1, 2a, 2b, and 3.

### Analysis of protein levels by Western blot

**Figure 6:**
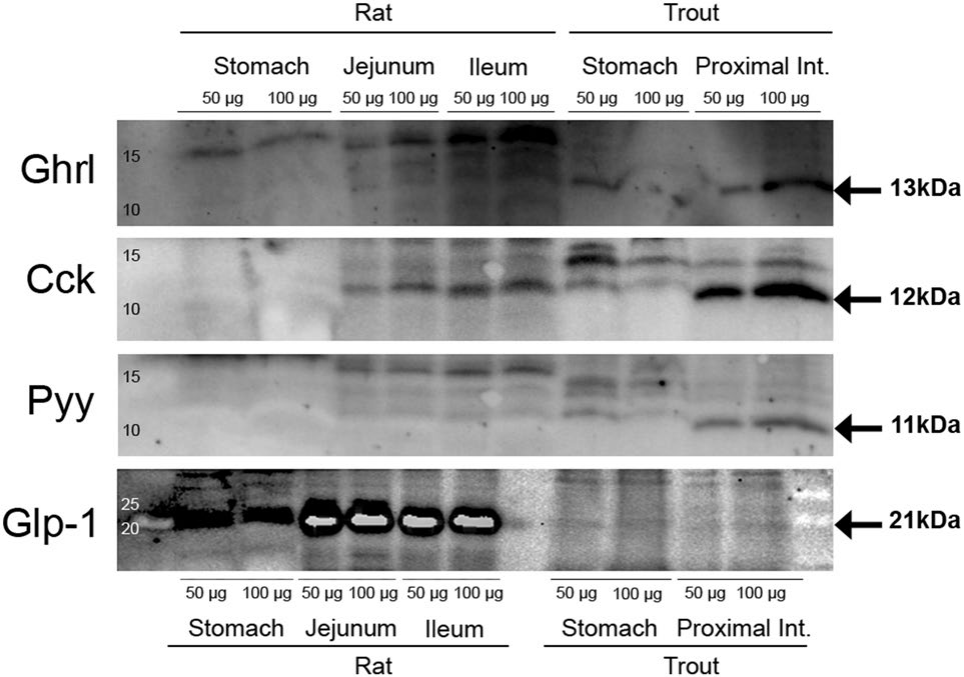
Western blot validation of selected antibodies for Ghrelin, Cck, Pyy and Glp-1 in stomach and proximal intestine of rainbow trout compared to the same (or equivalent) tissue in rat. Different quantities of protein were loaded on the gel per lane to verify the linearity of the method. The bands of interest are indicated with an arrow. Entire blots are shown in Supplementary Fig. S5.

Samples of stomach and proximal intestine (20 mg; n = 6 fish) were homogenized in 1 mL of buffer containing 10 mM Tris–HCl, 150 mM NaCl, 1 mM EGTA, 1 mM EDTA (pH 7.4), 4 mM sodium pyrophosphate, 100 mM sodium fluoride, 2 mM sodium orthovanadate, 0.5% NP40-IGEPAL, 1% Triton X-100, and 1.02 mg mL^–1^ protease inhibitor cocktail (Sigma-Aldrich). To prevent protein denaturation, tubes were kept on ice during the homogenization process. Homogenates were centrifuged for 15 min (1000×*g*; 4 °C), and supernatants were again centrifuged for 30 min (20,000×*g*; 4 °C). The resulting supernatants were recovered and stored at – 80 °C until further analysis. First, the concentration of protein in each sample was determined using Bradford assay with bovine serum albumin as standard. Then, samples (each containing 50 µg protein) were mixed with 4× Laemmli buffer containing 0.2% 2-mercaptoethanol (Bio-Rad) and denatured at 95 ºC for 10 min. Subsequently, they were electrophoresed in 17.5% acrylamide gels and transferred to a 0.2 µm pore-size nitrocellulose membrane (Bio-Rad) using the Trans-Blot Turbo transfer system (Bio-Rad). Membranes were blocked in Pierce Protein-Free T20 (PBS) Blocking Buffer (ThermoFisher, Waltham, MA, USA) during 60 min, and then incubated overnight with specific primary antibody. Primary antibodies used were: anti-GHRL (1:500; Phoenix Pharmaceuticals, Karlsruhe, Germany, Catalog # H-031-31), anti-CCK (1:500; Abcam, Cambridge, UK, Catalog # ab27441), anti-PYY (1:500; Abcam, Catalog # 22663), and anti-GLP-1 (1:500; Abcam, Catalog # 26278). Antibodies were validated for use in rainbow trout GIT, as determined by band comparison between rainbow trout and rat tissues (see Fig. 6 and Supplementary Fig. S5). After washing, membranes were incubated with goat anti-rabbit IgG (H + L) HRP conjugate (Abcam, Catalog # ab205718) or goat anti-mouse IgG (H + L) HRP conjugate (Abcam, Catalog # ab205719), both diluted 1:2000. For protein visualization, the membrane was incubated in Clarity Western ECL substrate (Bio-Rad) and imaged in a ChemiDoc Touch imaging system (Bio-Rad). Protein bands were quantified by densitometry using Image Lab software, relative to the amount of total protein.

### Statistical analysis

Data were first checked for normality and homogeneity of variance, and, if any of these requirements failed, they were log-transformed and re-checked. Then, one-way ANOVA followed by Student–Newman–Keuls multiple comparison test was used to assess differences among experimental groups, which were considered statistically significant when p < 0.05. All analyses were carried out using SigmaPlot version 12.0 (Systat Software Inc., San Jose, CA, USA).

## Supplementary Information

Supplementary Information.
