# Supplementary material for: First evidence for the presence of amino acid sensing mechanisms in the fish gastrointestinal tract
Source: Sci Rep. 2021 Mar 2;11:4933. doi: 10.1038/s41598-021-84303-9 (PMC7925595; doi:10.1038/s41598-021-84303-9)
Supplement: Supplementary file 1 — Supplementary Information. [file 41598_2021_84303_MOESM1_ESM.pdf]

First evidence for the presence of amino acid sensing mechanisms in the fish gastrointestinal tract

Jessica Calo[1], Ayelén M. Blanco[1*], Sara Comesaña[1], Marta Conde-Sieira[1], Sofía Morais[2], José L. Soengas[1]

[1] Laboratorio de Fisioloxía Animal, Departamento de Bioloxía Funcional e Ciencias da Saúde, Facultade de Bioloxía and Centro de Investigación Mariña-CIM, Universidade de Vigo, Vigo, Pontevedra, Spain.

[2] Lucta, Innovation Division, Autonomous University of Barcelona Research Park, Bellaterra, Spain

*** Corresponding author:** Ayelén M. Blanco (amblanco@uvigo.es)

ORCID: 0000-0002-7683-4154

Supplementary Fig. S1. Full-length images of agarose gels included in Figures 1 and 4. Molecular markers are included. See corresponding figure legends for details.

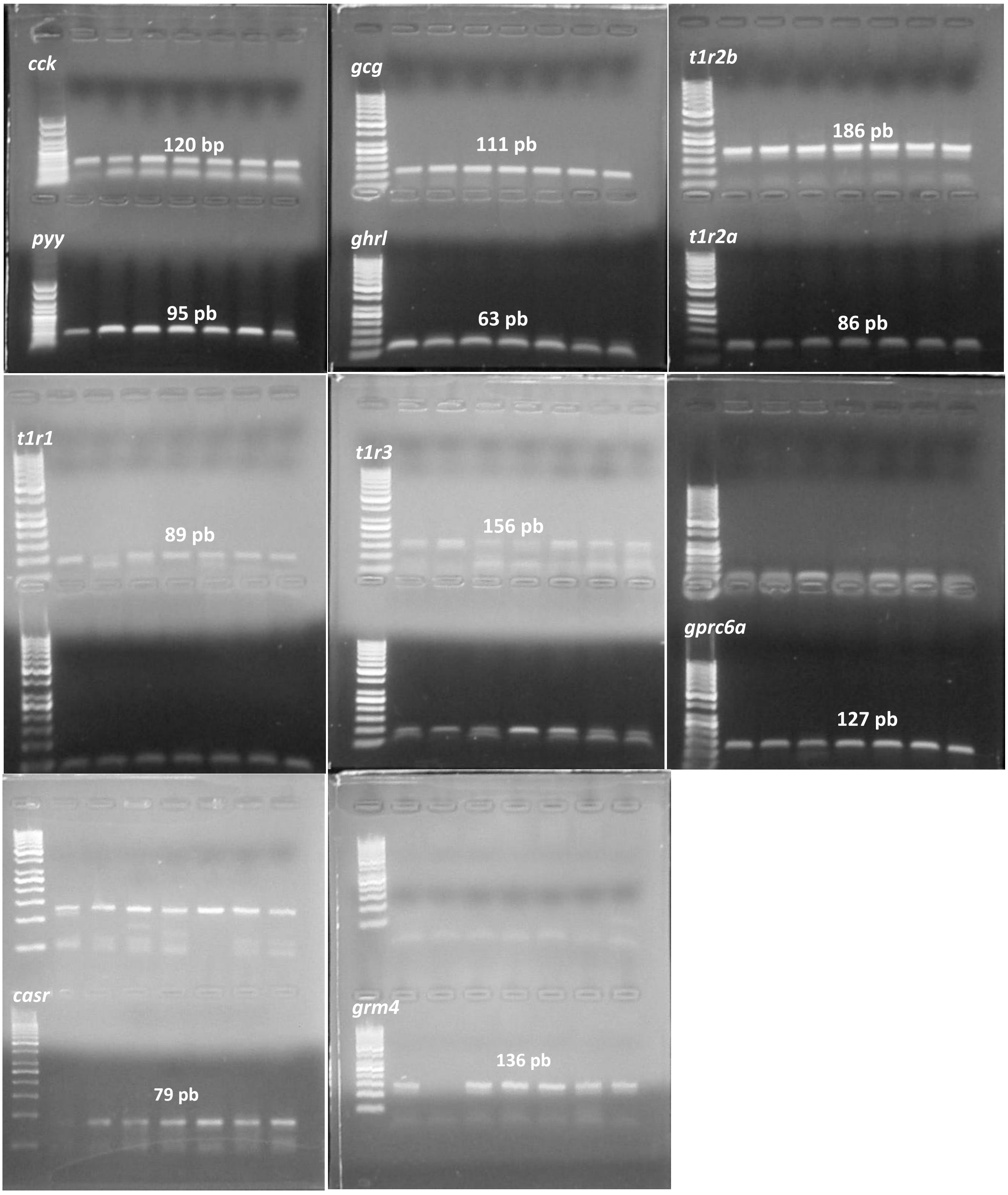

Supplementary Fig. S2. Full-length images of Western blots included in Figure 5 (e – h). Bands shown in Figure 5 (e – h) are boxed. For each protein, two separate blots containing in total of six samples per treatment were performed. Molecular markers are included. See corresponding figure legend for details.

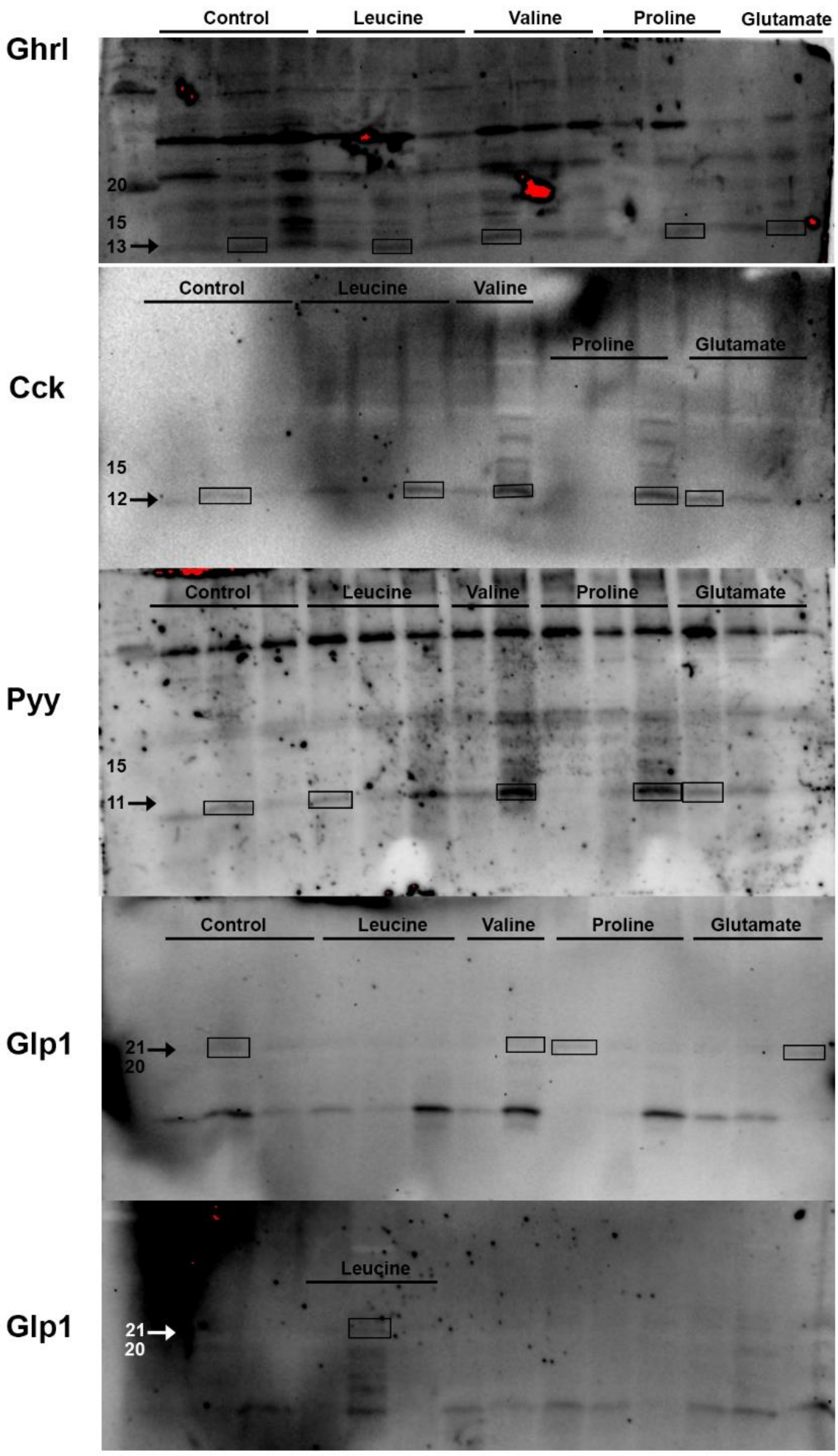

Supplementary Fig. S3. Effects of intragastric administration of 40 µmol/mL of L-leucine, L-valine, L-proline or L-glutamate on the plasma levels of lactate (a), glucose (b) and α-amino acids (c) in rainbow trout. Data are expressed as mean + SEM (n = 12). Statistical differences among groups were assessed by one-way ANOVA and post-hoc Student-Newman-Keuls test. Significant differences are denoted by the use of letters: bars sharing the same letter are not statistically significant, while if two bars have different letters they are significantly different (p<0.05).

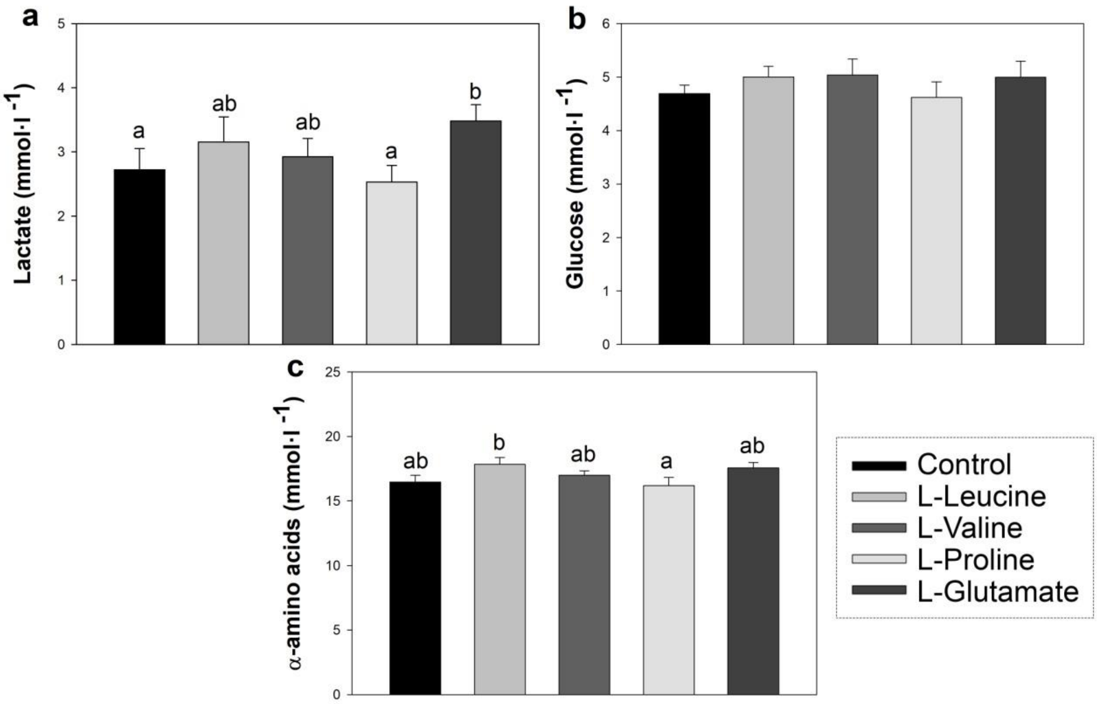

Supplementary Fig. S4. Schematic representation of the rainbow trout gastrointestinal tract, showing the regions in which it was divided for the experiments carried out in this study. The areas sampled within each region are squared.

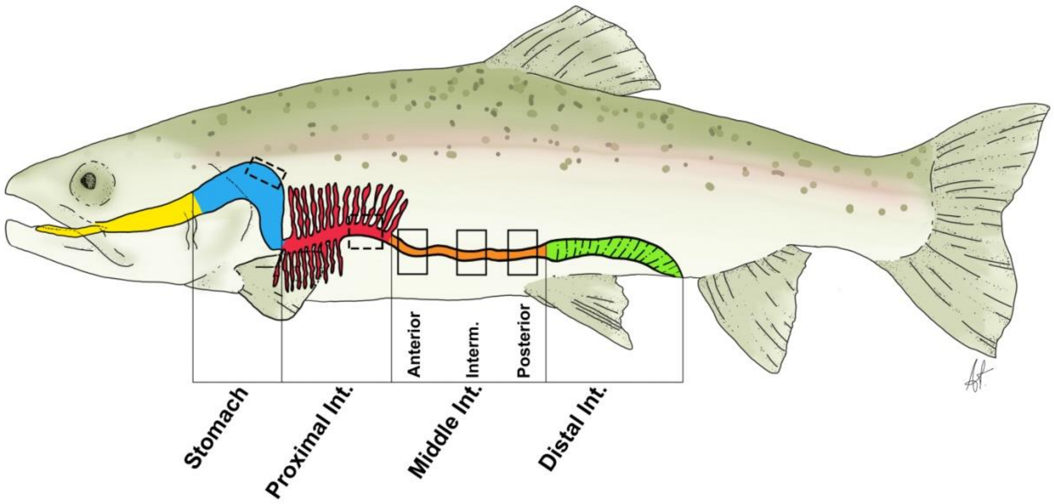

Supplementary Fig. S5. Full-length images of Western blots included in Figure 6. Molecular markers are included. See corresponding figure legend for details.

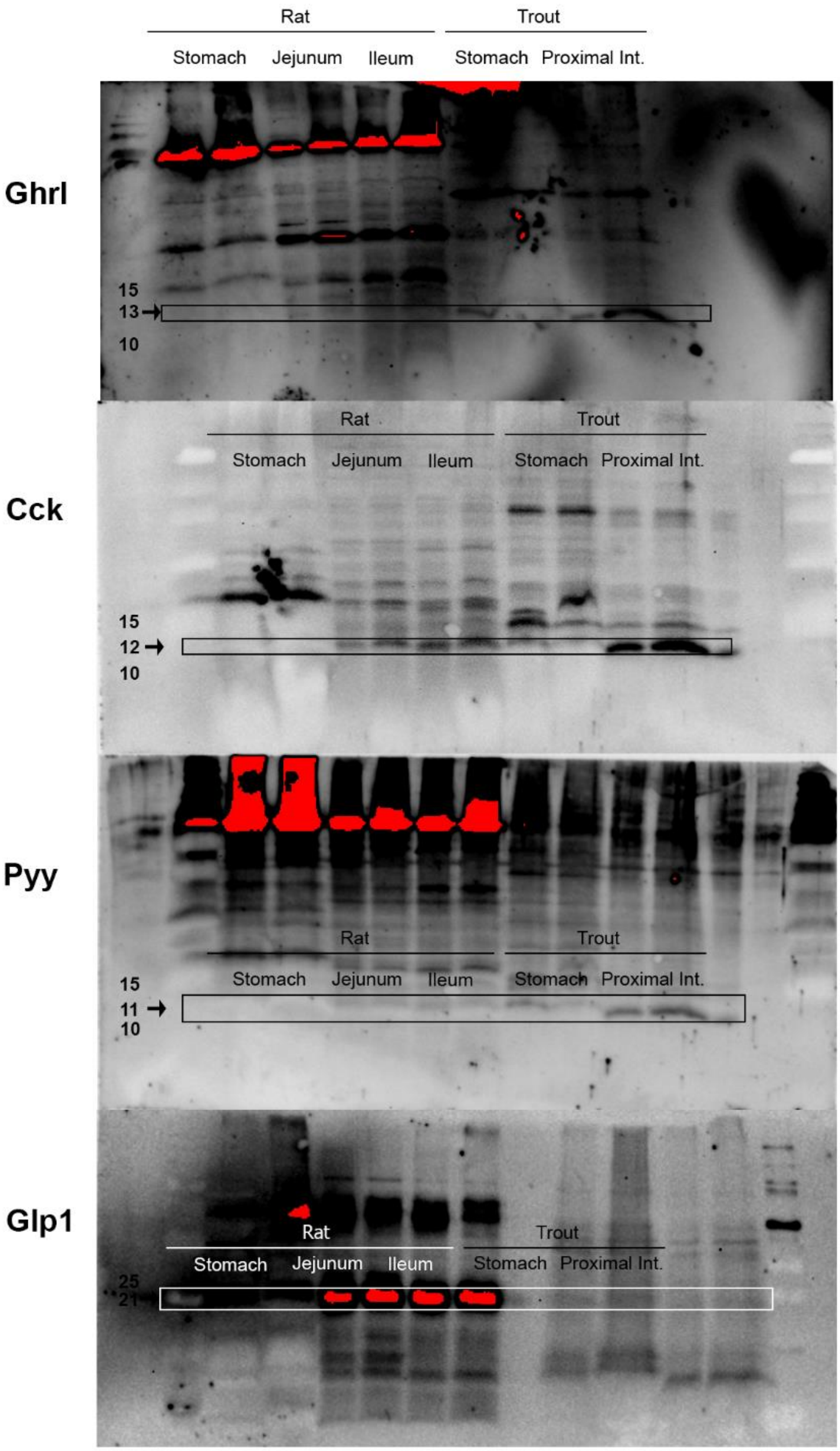

Supplemental Table S1. List of primers used during attempts to amplify *grm1*, *plcβ2* and *trpm5* in the gastrointestinal tract of rainbow trout.

Gene	GenBank accession number	Forward primer (5' to 3')	Reverse primer (5' to 3')
grm1	XM_021600739.1	CCCTGGAGCAGAGCATTGAGT	CTTGGAGCCGTCCTTGTCATC
		GCAGTACCGCTTCCAGTGCC	TGCCTAGATCACCGGAGCAG
	XM_021583050.1	AGCTGTTGCTGGATCTGTACGC	CTCGTTGAAGTTGGCCGGG
		GTTGTGCCCTCTGACACTCTC	TCTTGTCTGAATGGGCGATAC
		GGAGGTGACGTCTTTTGATGA	GGGTGAGCTGGACACTGACTA
	XM_021600741.1	ATGGGCAGATCGTGATGAGGT	TTCCGTGAGTTGGTGTCCAAA
		CAGGACAGTAAGATGGGCTTCG	TGAGCAGGTAATCCAGCAGCTT
		GCGTTTGGACACCAACTCAC	ATTGCCTAGATCACCGGAGC
		TGCTCCGGTGATCTAGGCAA	ACATCCTCCCCTGATACCCC
	XM_021573769.1	CCGCCTCAACACCAACACCA	CCCATCTTACTGTCCTGGAC
plcb2	XM_021584705.1	GGATTGCTGGAAGGGAAAACC	CGGGGTACTGTGACGTCTTGA
		TGCCAGATGGTGGCGCTCAA	CACCGCTCCAGAATGTGCCTT
		GCTGCCTGGAGCTGGATTGC	CTTGAAGGCGCTTTCCGCGA
		TGGCGCTCAACTACCAAAC	CTTCTTGTCACTACGGCGGA
		CCAGAGCCTGAACCCAACAT	AGGGGTCAACTCTGTGGCTA
		TCACTTCCTGTTGATCTGCGG	AGCATGTCTACCCCAGGAAG
trpm5	XM_021586867.1	GCCAGAGTCAGGAAGCTCAGG	TGTGGCTCTTAGCGATGTCCA
		CAGGGCAACACAGACATCTTCT	CCAGTTTGGCCAGATAGTTCTCC
		ACTTTGCCTTCCTCATCCTGT	GTTCCAGTTGTCCTCCACGTA

grm1, metabotropic glutamate receptor 1; *plcb2*, phospholipase C β2; *trpm5*, transient receptor potential cation channel subfamily M member 5
